# On the relationship between an Asian haplotype on chromosome 6 that reduces androstenone levels in boars and the differential expression of *SULT2A1* in the testis

**DOI:** 10.1186/1471-2156-15-4

**Published:** 2014-01-09

**Authors:** André M Hidalgo, John WM Bastiaansen, Barbara Harlizius, Hendrik-Jan Megens, Ole Madsen, Richard PMA Crooijmans, Martien AM Groenen

**Affiliations:** 1 Animal Breeding and Genomics Centre, Wageningen University, Wageningen, the Netherlands; 2 Department of Animal Breeding and Genetics, Swedish University of Agricultural Sciences, Uppsala, Sweden; 3 TOPIGS Research Center IPG, Beuningen, the Netherlands

**Keywords:** Asian haplotype, Boar taint, RNA-seq, SSC6, Whole genome sequencing

## Abstract

**Background:**

Androstenone is one of the major compounds responsible for boar taint, a pronounced urine-like odor produced when cooking boar meat. Several studies have identified quantitative trait loci (QTL) for androstenone level on *Sus scrofa* chromosome (SSC) 6. For one of the candidate genes in the region *SULT2A1*, a difference in expression levels in the testis has been shown at the protein and RNA level.

**Results:**

Haplotypes were predicted for the QTL region and their effects were estimated showing that haplotype 1 was consistently related with a lower level, and haplotype 2 with a higher level of androstenone. A recombinant haplotype allowed us to narrow down the QTL region from 3.75 Mbp to 1.94 Mbp. An RNA-seq analysis of the liver and testis revealed six genes that were differentially expressed between homozygotes of haplotypes 1 and 2. Genomic sequences of these differentially expressed genes were checked for variations within potential regulatory regions. We identified one variant located within a CpG island that could affect expression of *SULT2A1* gene. An allele-specific expression analysis in the testis did not show differential expression between the alleles of *SULT2A1* located on the different haplotypes in heterozygous animals. However a synonymous mutation C166T (SSC6: 49,117,861 bp in Sscrofa 10.2; *C/T*) was identified within the exon 2 of *SULT2A1* for which the haplotype 2 only had the *C* allele which was higher expressed than the *T* allele, indicating haplotype-independent allelic-imbalanced expression between the two alleles. A phylogenetic analysis for the 1.94 Mbp region revealed that haplotype 1, associated with low androstenone level, originated from Asia.

**Conclusions:**

Differential expression could be observed for six genes by RNA-seq analysis. No difference in the ratio of *C*:*T* expression of *SULT2A1* for the haplotypes was found by the allele-specific expression analysis, however, a difference in expression between the *C* over *T* allele was found for a variation within *SULT2A1*, showing that the difference in androstenone levels between the haplotypes is not caused by the SNP in exon 2.

## Background

Androstenone (5α-androst-16-en-3-one) is a steroid hormone synthesized in the Leydig cells of the testis in a stepwise conversion involving 3β-hydroxysteroid dehydrogenase (HSD) and 5α-reductase enzymes [1]. In pigs, androstenone acts as a sex pheromone which attracts female pigs making them more receptive to mating [2]. Androstenone is degraded in the liver and salivary gland by 3α-HSD enzymes resulting in α-androstenol and by 3β-HSD enzymes resulting in β-androstenol [1,3]. Sulfoconjugated androstenols are eliminated mainly in the urine and bile. Androstenone is one of the major compounds responsible for boar taint, a pronounced urine-like odor produced when cooking meat from intact male pigs, or boar meat [4]. As unconjugated androstenone and androstenol are the forms that most easily accumulate in adipose tissue and hereby lead to boar taint [5], conjugation plays an important role in the prevention of boar taint. At high concentrations in the fat, androstenone influences consumer acceptability of pork [6]. In current breeding practice, castration of male piglets is used to prevent the boar taint. Castration, however, is undesirable not only for technical reasons, as castrated male pigs have fatter carcasses and reduced feed efficiency [7], but also because of animal welfare concerns and future legislation restriction. Therefore, the development of an alternative to castration is needed.

The development of a medium-density 60 K porcine single nucleotide polymorphism (SNP) chip [8], has enabled genome-wide association studies (GWAS) to efficiently map regions throughout the genome affecting phenotypic traits such as the androstenone level. While GWAS can identify significant marker associations, the current SNP density on the Illumina PorcineSNP60 BeadChip often leads to clusters of markers covering a region that is still too large to allow accurate identification of the responsible genes or variants. Hence, there is still the need to reduce the size of these clusters if the aim is to find causative relations between gene(s) or variants that affect phenotypic traits like androstenone level in fat.

Several studies [9-12] have identified quantitative trait loci (QTL) for androstenone level on *Sus scrofa* chromosome (SSC) 6. Duijvesteijn et al. [9] performed a GWAS unveiling an 8 Mbp region on SSC6 associated with androstenone level in boars of a Duroc-based population. Similarly, Grindflek et al. [10] reported a QTL for Duroc animals within a 7.1 Mbp region that overlaps with the region found by Duijvesteijn et al. [9]. Within the QTL region on SSC6, Duijvesteijn et al. [9] showed that there are haplotypes related to low and high average levels of androstenone in fat. Expression studies that compared boars with low- and high-androstenone level [13-15] found differential expression of several genes including sulfotransferase family 2A dehydroepiandrosterone-preferring member 1 (*SULT2A1*). *SULT2A1* is located within the QTL region and is a strong candidate gene to have an effect on androstenone level. Since QTL regions are large, fine mapping studies need to be carried out to identify causative variants and to enable the use of these QTL in breeding programs.

The goal of this study was to narrow down the QTL region on SSC6 previously reported by Duijvesteijn et al. [9], to identify and characterize genes and SNP variants that affect androstenone level in pigs, and to determine whether the effects of low- and high-androstenone haplotypes are caused by differential expression of *SULT2A1*.

## Results

**Figure 1:**
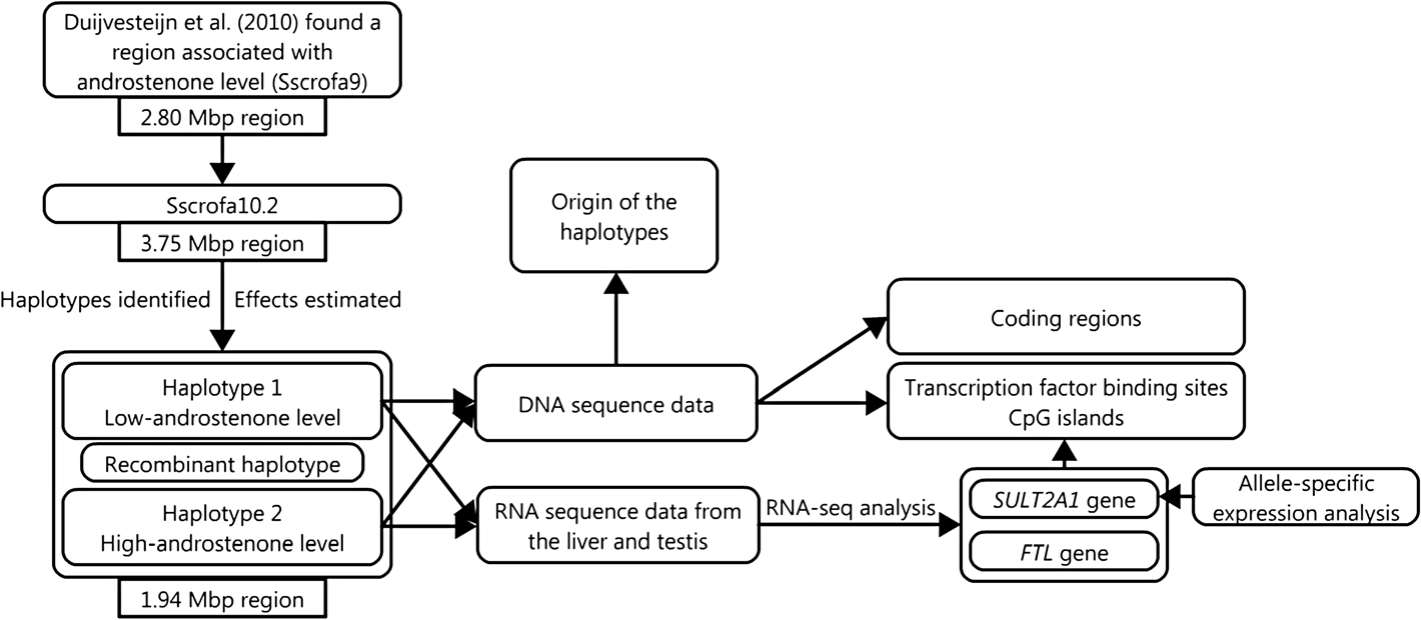
**Flowchart of the steps taken in the current study.** We started the analysis with a region associated with androstenone level previously found on SSC6. The position of the markers were adjusted to the Sscrofa10.2 assembly of the reference genome. Afterwards, haplotypes for the region were identified and their effects were estimated. The region was narrowed down using information from a recombinant haplotype. RNA-seq analysis, within the narrowed region, was performed in the liver and testis. For candidate genes that were differentially expressed, we used whole genome sequence data to look for variations within regulatory regions and also to look for variations within coding regions for all genes within the narrowed region. Allele-specific expression analysis in the testis was performed for *SULT2A1* gene because a variation is located within a regulatory region. Finally, we analyzed genomic sequence data to assess the origin of the haplotypes.

Androstenone level was obtained from 2,750 boars that belonged to six purebred populations and five crosses. The flowchart (Figure 1) provides an overview of the study to clarify the steps that were taken in association mapping, whole genome sequencing, and functional analyses.

### Region of interest and haplotypes

Markers associated with androstenone were identified by Duijvesteijn et al. [9] in the region from position 36,907,969 bp to 44,939,360 bp on SSC6 using the Sscrofa9 assembly of the reference genome. A target region of 2.8 Mbp, from position 36,907,969 bp to 39,697,649 bp, containing the peak associations, was defined by Duijvesteijn et al. [9] and was used in our study. The present study used the Sscrofa10.2 assembly of the reference genome [16], in which the 2.8 Mbp region in genome build Sscrofa9 corresponded to a 3.75 Mbp region on SSC6, from position 48,585,961 bp to 52,336,598 bp. This region contained two linkage disequilibrium (LD) blocks with a total of 46 markers on the Illumina PorcineSNP60 BeadChip that were polymorphic in our study (Additional file 1), 29 of which are significant for androstenone level, identical to the ones reported by Duijvesteijn et al. [9]. Prediction of the haplotypes for the 46 SNPs across populations revealed 10 haplotypes with a frequency above 1%.

### Effects and association analysis of haplotypes

**Figure 2:**
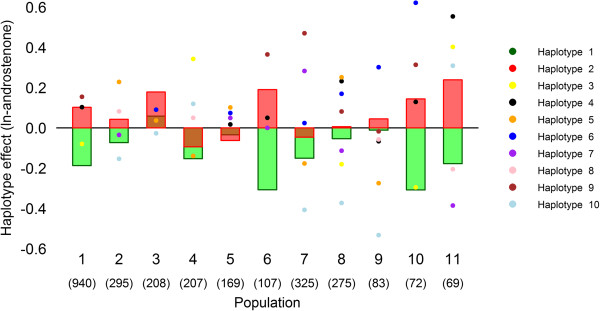
**Haplotype effects across all populations.** Effects estimated for the haplotypes 1 and 2 (bars) and eight other haplotypes (dots) across 11 populations (number of animals).

Effects on androstenone level were estimated for the ten haplotypes (Additional file 2). Haplotypes 1 and 2 were present in all populations; haplotype 1 was consistently related with a lower level, and haplotype 2 with a higher level of androstenone (Figure 2).

**Figure 3:**
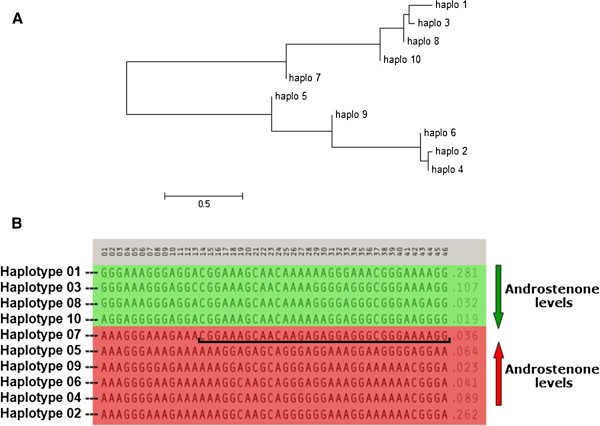
**Phylogeny of the 10 haplotypes. (A)** Phylogenetic tree for the 10 most frequent haplotypes and **(B)** the 10 most frequent haplotypes across all populations ordered according to the phylogenetic tree and colored according to their effect on androstenone level. The underlined region indicates similarity between haplotype 7 and low-androstenone haplotypes.

A phylogenetic tree was constructed using MEGA 5 [17] based on similarities among the 46 SNPs of the haplotypes (Figure 3A). Haplotypes were arranged in two groups, with haplotypes 1, 3, 7, 8, and 10 forming one group and haplotypes 2, 4, 5, 6, and 9 another group. To determine whether the relation of haplotypes 1 and 2 to androstenone level followed the phylogenetic division, we analyzed the association between haplotypes and androstenone using Treescan [18] which “cuts” the phylogenetic tree at different branches and tests whether the groups created by the cut are statistically different in their effect on the phenotype. Dividing the tree between haplotypes 1, 3, 8, 10, and 7, 5, 9, 6, 2, 4; or between haplotypes 1, 3, 8, 10, 7, and 5, 9, 6, 2, 4 resulted in statistically significant differences (P < 0.0001) and explained the largest proportion of phenotypic variation (0.049 and 0.051, respectively).

Haplotype 7 is a recombinant haplotype: the region from SNP 1 to 13 is similar to haplotype 2 (high androstenone level), whereas the region from SNP 14 to 46 is similar to haplotype 1 (low androstenone level) (Figure 3B). Therefore, with a posterior analysis, the effects of haplotypes 1, 2, and 7 were estimated using only populations in which haplotype 7 is segregating (N = 1240). The effect of haplotype 7 (effect = 0.05) is significantly different from haplotype 1 (effect = −0.16), whereas it is not significantly different from haplotype 2 (effect = 0.00). The effect of haplotype 7 could therefore be grouped together with haplotype 2, indicating that the region from haplotype 7 that is similar to haplotype 2 causes the effect. This allowed us to narrow down the associated region from 3.75 Mbp (48,585,961 bp - 52,336,598 bp) to 1.94 Mbp (48,317,509 bp – 50,259,057 bp). When testing the remaining haplotypes 3, 4, 5, 6, 8, 9, and 10 in the same way, their effects were all congruent with the expectation, except for the rare haplotypes 8 and 10 which were not significantly different from either haplotypes 1 or 2 (Additional file 3).

### RNA-seq analysis

To determine whether genes within the narrowed region were differentially expressed between haplotypes 1 and 2, an RNA-seq analysis was performed in the liver and testis. A “haplotype-1 pool” was made from four animals homozygous for haplotype 1, and a “haplotype-2 pool” from four animals homozygous for haplotype 2. After the rearrangement of the map in the new reference genome assembly build10.2 it was found that the haplotype-2 pool contained six copies of haplotype 2 and two copies that were haplotype-2-like (rare haplotypes that differed from haplotype 2 in three positions).

A total of 79 genes were located within the narrowed region. Among these, three genes (4%) were found to be differentially expressed in the liver: sperm acrosome membrane-associated protein 4 (*SPACA4*), synaptogyrin 4 (*SYNGR4*), tubby-like protein 2 (*TULP2*); and three genes (4%) in the testis: ferritin light polypeptide (*FTL*), glioma tumor suppressor candidate region gene 1 (*GLTSCR1*), and sulfotransferase family 2A dehydroepiandrosterone-preferring member 1 (*SULT2A1*) (Table 1).

**Table 1:** Results for genes differentially expressed in the liver and testis between pools of animals homozygous for low- and high-androstenone haplotypes

**Gene**	**Location**	**Haplo-1 Pool**	**Haplo-2 Pool**	Log2 FC*	**P value**
Liver					
SYNGR4	49625413-49627375	0.71	7.81	3.47	3.2 x10^-4^
SPACA4	49711175-49719812	2.00	11.76	2.56	2.21 x10^-3^
TULP2	50142849-50151782	0.67	6.45	3.27	6.72 x10^-6^
Testis					
GLTSCR1	48928610-48946950	4.39	1.88	−1.22	1.34 x10^-6^
SULT2A1	49108566-49119941	40.06	104.83	1.39	1.41 x10^-7^
FTL	50078375-50097694	782.79	1618.46	1.05	1.95 x10^-5^

*Fold changes (FC) are calculated relative to low-androstenone haplotypes, hence indicate the times of up-regulation in the high-androstenone haplotype compared to the low-androstenone haplotype.

### Functional analysis

Whole-genome sequencing data were used to investigate the complete set of SNP variants in the narrowed region. From the 55 animals for which the whole genome sequencing data were available (see M&M for details), 10 animals were homozygous for either haplotype 1 or 2 according to the 13 SNPs located within the 1.94 Mbp interval. An animal was considered to be homozygous when it met two criteria: (1) average heterozygosity for the narrowed region was very low (Additional file 4), (2) the genotypes of the 13 SNPs overlapping with the Illumina PorcineSNP60 BeadChip in the region were identical to the sequencing data.

#### Coding regions

From the genome sequencing data, 1,897 single nucleotide differences were found between haplotypes 1 and 2 in the 1.94 Mbp interval. To detect functional genetic variants between the haplotypes, differences were annotated using ANNOVAR [19]. Of the 1,897 SNPs, 75 (3.95%) were located in exonic regions, with 17 of them being non-synonymous and 58 being synonymous variations (Additional file 5). Within the five genes previously suggested as candidates for effects on androstenone by Duijvesteijn et al. and Grindflek et al. [9,13], we found three synonymous and one non-synonymous variation. Gene *SULT2A1* contained one synonymous variation (C/T) within exon 2 at position 166 (SSC6: 49,117,861 bp in Sscfrofa10.2); Hydroxysteroid (17-beta) dehydrogenase 14 (*HSDB17B14*) contained a non-synonymous variation (T/G) within exon 4 at position 217 (SSC6: 49,889,443 bp in Sscfrofa10.2); Lutropin subunit beta (*LHB*) contained a synonymous variation (C/T) within exon 1 at position 147 (SSC6: 50,064,434 bp in Sscfrofa10.2); *FTL* contained a synonymous variation (C/G) within exon 4 at position 474 (SSC6: 50,096,119 bp in Sscrofa10.2); and for sulfotransferase family cytosolic, 2B member 1 (*SULT2B1*) no nucleotide variation was found in any of the exons.

The impact of the non-synonymous variations was assessed using PolyPhen2 [20], which showed that the T/G variation in *HSDB17B14* was unlikely to affect the function of the protein. Regarding other genes within the 1.94 Mbp interval, that have non-synonymous variations but are not considered as a candidate gene, a variation within exon 1 of fucosyltransferase 1 (*FUT1*) is probably damaging the functionality of the protein (PSIC score: 2.092). Probably-damaging status indicates that the variation is, with high confidence, expected to affect protein function.

#### Regulatory regions

Because *SULT2A1* and *FTL* were differentially expressed between pools of haplotype 1 and haplotype 2 animals and are functional candidate genes, their up and downstream sequences (±2,000) were examined for the presence of potential transcription factor binding sites (TFBS) that were conserved across three species (*Sus scrofa*, *Bos taurus*, and *Homo sapiens*). None of the fixed differences between haplotypes 1 and 2 were located within predicted TFBS. In addition to the absence of single nucleotide differences between haplotypes 1 and 2 within the TFBS, we checked the overlap of the TFBS with copy number variations (CNVs) identified by Paudel et al. [21]. No CNVs were identified that could affect TFBS near *SULT2A1* and *FTL* genes.

CpG islands were predicted for the *SULT2A1* and *FTL* genes including their up and downstream sequence (±2000 bp). Two CpG islands of at least 200 bp, 50% of GC content, and 60% of average observed-to-expected ratio of *C* plus *G* were detected for each of the genes. One of the four CpG islands (49,110,687 bp - 49,110,889 bp) which were predicted for *SULT2A1* contained a SNP (C/G, 49,110,873 bp). This CpG island had 18 CpGs. None of the identified CpG islands contained CNVs.

### Validation of differential *SULT2A1* expression in the testis

Of the genes that were differentially expressed between the low- and high-androstenone pools, *SULT2A1*, based on its function*,* is a particularly strong positional candidate gene. To validate the difference in expression in the testis found between the haplotype-1 and haplotype-2 pools, we made use of a synonymous SNP (C/T; SSC6: 49,117,861 bp in Sscfrofa10.2 – see details below on detection of this SNP) within exon 2 of the *SULT2A1* gene described by Sinclair et al. [22]. The C/T variation was not in complete LD with the low- and high-androstenone haplotypes. The *T* allele had a frequency of 0.34 and was only found on the haplotype 1, the *C* allele was found on both low and high androstenone haplotypes (2, 3, and 4). It allowed for a comparison of not only the ratio of *C*:*T* expression between low- and high-androstenone haplotypes but also between the *SULT2A1* alleles. The QTL effects of the *C* and *T* alleles were investigated and both the *C* and *T* alleles that were located on low-androstenone haplotypes were significantly different from *C* alleles that were located on high-androstenone haplotypes while within the low-androstenone haplotypes the *C* and *T* alleles were not different (Additional file 6). To certify that the allele-specific expression analysis was sensitive enough to detect the expression differences found in the RNA-seq data, genomic DNA from two animals, homozygous for the *C* or *T* allele, were mixed in seven ratios and in three concentrations. The result of this analysis showed high sensitivity to distinguish between the expected difference in expression levels and a strong linear relation between the observed and expected ratios (R^2^ in three concentrations: 2.5 ng = 0.93; 10 ng = 0.72; 40 ng = 0.97) (Additional file 7).

**Figure 4:**
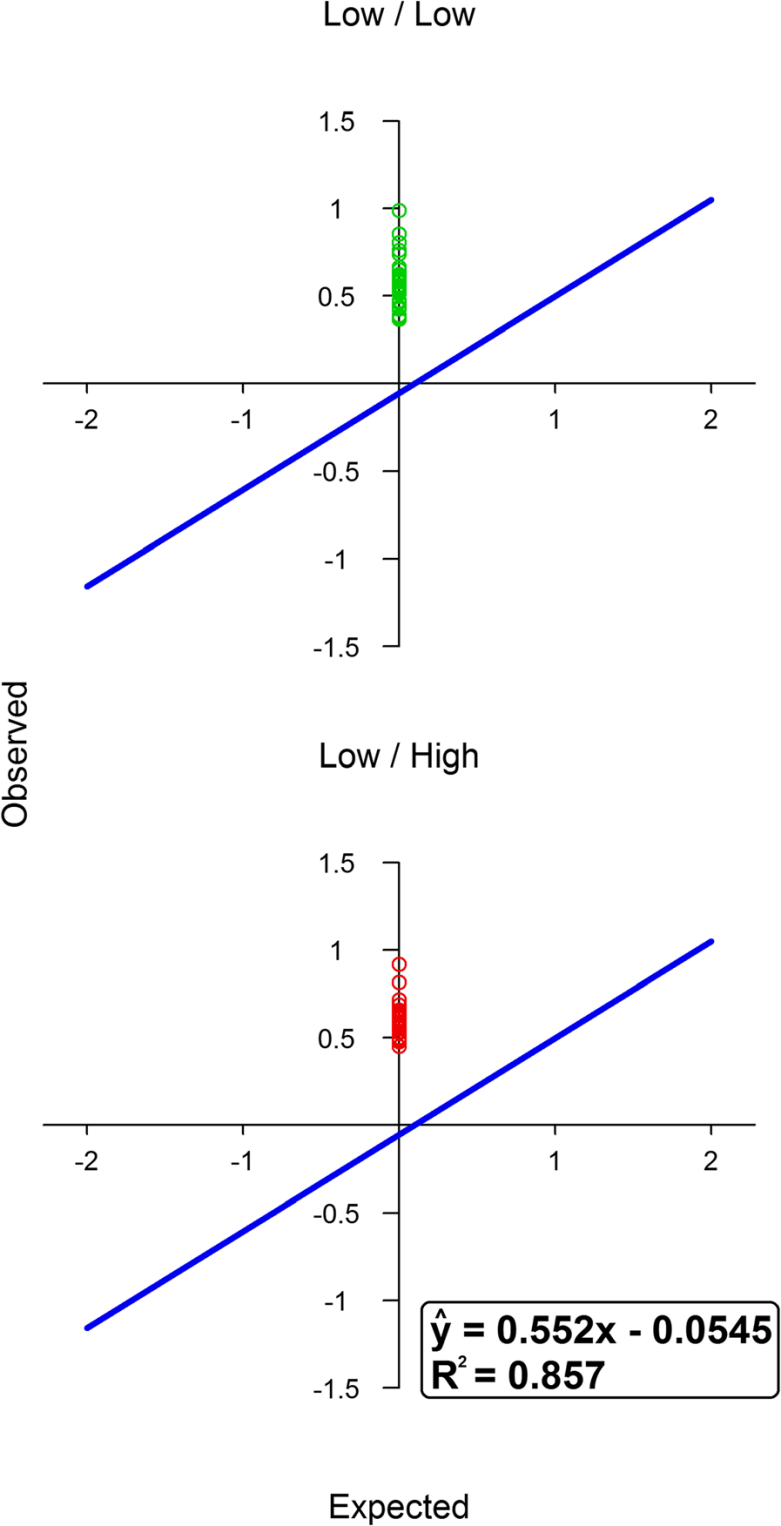
**Allele-specific expression analysis.** Ratios of *C:T* cDNA expression levels in testis from heterozygous animals for exonic variation in *SULT2A1*. Heterozygous animals’ diplotypes are indicated in the titles (low/low, low/high). Standard curve fitted to all control samples (blue line) and its regression equation and coefficient of determination are shown.

Heterozygous animals (C/T) were one of two different androstenone diplotypes (low/low or high/low) but there was no difference in the ratio of *C*:*T* expression between the low/low and high/low diplotype. A difference in expression was however observed between the *C* and *T* alleles of *SULT2A1* with the *C* allele always being higher expressed than the *T* allele (ratio *C:T* = 1.5:1, s.d. = 0.13, Figure 4). The mean ratio of 1.5:1 was calculated based on the 67 heterozygous animals studied in the allele-specific expression analysis. Genotyping of this SNP on the animals from the pools used in the RNA-seq analysis showed that the haplotype-1 pool contained 4*C* and 4*T* alleles whereas the haplotype-2 pool contained only *C* alleles. Thus, the observed difference in expression between haplotype-1 and haplotype-2 pools in *SULT2A1* expression could be related to differences in expression of the *C/T* alleles in exon 2.

### Origin of the haplotypes

**Figure 5:**
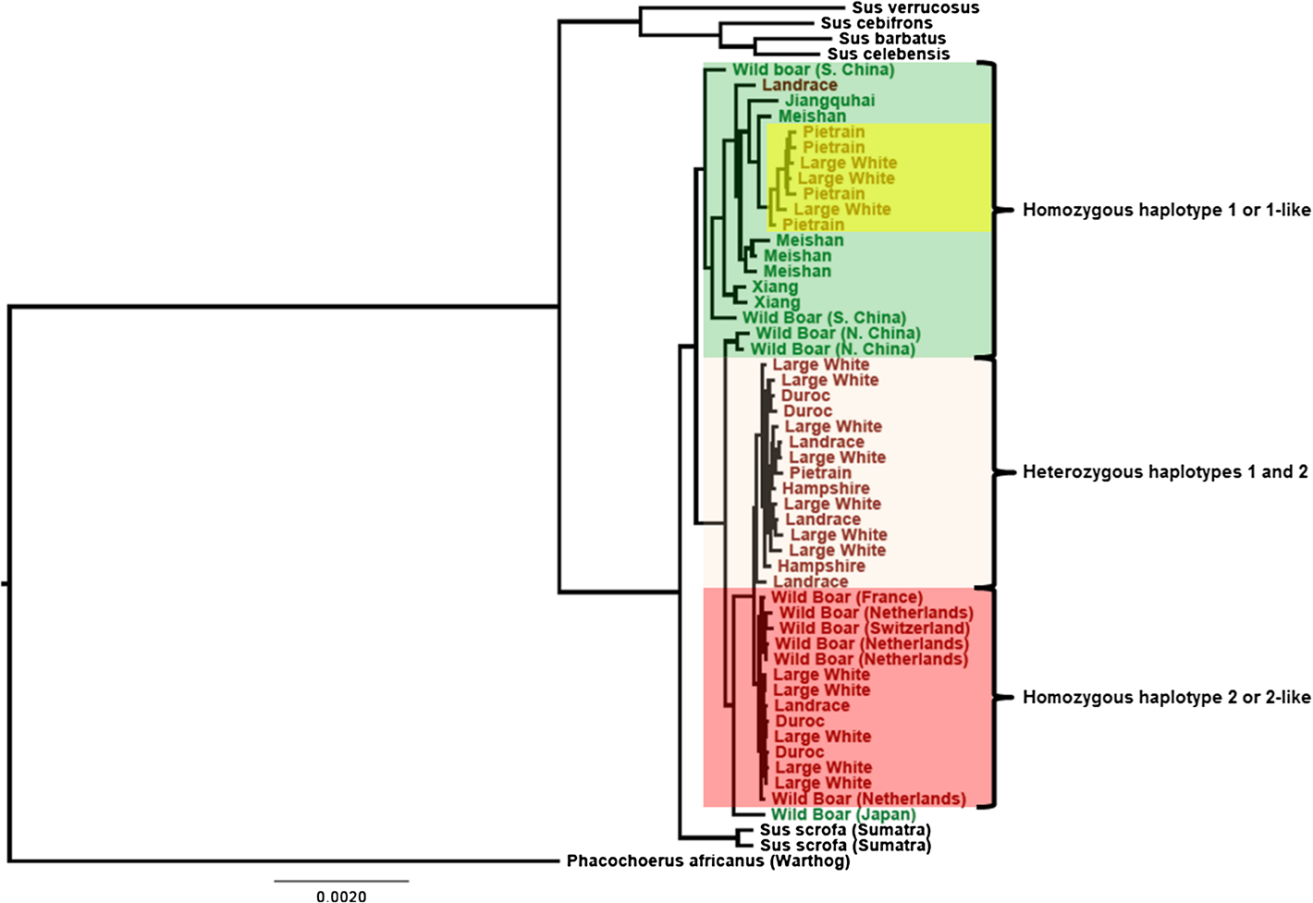
**Phylogenetic tree for the haplotypes within the narrowed region in whole genome sequenced animals.** Asian animals (green) are homozygous for haplotype 1 or 1-like, whereas European animals (red) have both haplotypes. European cluster of animals within the Asian animals group (yellow) shows that the haplotype 1 (low androstenone) originated from Asian breeds.

A phylogenetic analysis was applied to investigate the origin of the haplotypes by extracting the 1.94 Mbp region from sequencing data from the 55 sequenced animals (Figure 5) [23].

The clustering of animals revealed by the phylogenetic tree computed using sequencing data was concordant with the tree based on the haplotypes from this region computed from the Illumina PorcineSNP60 BeadChip data. Haplotypes were grouped into three clusters: animals homozygous for haplotype 1 or haplotypes 1-like, animals homozygous for haplotype 2 or haplotypes 2-like, and animals heterozygous for haplotypes 1 and 2. Among the Asian animals only haplotype 1 or haplotypes 1-like were found, whereas in European wild boars only haplotype 2 or haplotypes 2-like were found. On the other hand, commercial European breeds are located within all three groups, showing that those animals carry all haplotypes.

## Discussion

The SSC6 region associated with androstenone level was reduced from 3.75 Mbp to 1.94 Mbp and the association of haplotypes in the region with androstenone was replicated in independent populations. Haplotype 1 reduces the androstenone level across populations and can be potentially implemented in marker-assisted selection by pig breeding companies. Selection for haplotype 1 would speed up the genetic response for lower androstenone level, which would reduce the incidence of boar taint, countering the effects of international policies regarding castration of piglets. The association of *SULT2A1* expression in the testis with the level of androstenone [13-15] was confirmed by sequence analysis of RNA pools. Validation of differential expression showed that a SNP located within exon 2 of *SULT2A1* presented higher expression of the *C* over the *T* allele, confirming the result from the RNA-seq analysis and suggesting allelic-imbalanced expression of the two alleles. This difference in the ratio of *C*:*T* is however not associated with the haplotypes. A thorough search for functional SNP variation was carried out and resulted in a limited number of non-synonymous variants, despite the very high density of genes in the region.

### Region of interest and haplotypes

The number of SNPs within the region of interest is higher in our study compared to Duijvesteijn et al. [9], due to the improved assembly of the reference genome Sscrofa10.2. Non-associated SNPs that were previously located within the associated region were moved elsewhere on the genome; simultaneously, additional non-associated SNPs were now included in the associated region.

Predicted haplotypes varied in number and frequency among the 11 populations. A greater number of haplotypes were found in those populations that represent crosses of purebred populations (populations 7 to 11). This was expected as crosses are made between divergent purebred populations that have different frequencies of haplotypes. Crosses will therefore combine haplotypes present in the purebred populations.

### Effects and association analysis of haplotypes

Across all populations, haplotype 1 was consistently related with lower levels, and haplotype 2 with higher levels of androstenone. The haplotype tree showed two very distinct groups of haplotypes. When this tree was used to detect associations between (groups of) haplotypes and phenotypes, the estimated effects from regression analyses were in good agreement with the evolutionary history of the haplotypes. Haplotypes similar in sequence to haplotype 1 also have similar effects, decreasing androstenone, and haplotypes similar to haplotype 2 have effects that increase androstenone.

After confirming that in general, haplotypes similar to 1 are associated with low and haplotypes similar to 2 are associated with high androstenone level, a posterior analysis using these two haplotypes together with the recombinant haplotype 7, placed haplotype 7 in the high-androstenone group. This placement was important because the haplotype 7 sequence is a recombination between haplotypes 1 and 2. From this result it was possible to deduce that the region from SNP 1 to 13 harbors the genetic variation responsible for the QTL for androstenone level in boars. Because it is unknown where the recombination took place the region was defined including the flanking intervals, 3′ up to SNP 14, and 5′ up to the next SNP outside the LD block (SSC6: 48,317,509 bp – 50,259,057 bp, between genes *SAE1* and *SLC17A7*). The assignment of haplotype 7 allowed us to narrow down the associated region from 3.75 Mbp to 1.94 Mbp.

This region is very gene-dense and contains several candidate genes for androstenone-level QTL [9,13]: *SULT2A1*, *SULT2B1*, *HSD17B14*, *LHB*, and *FTL*. The region is only ~0.3 cM long and has a low recombination rate [24] (Additional file 8). This is consistent with the low number of haplotypes identified within this region, even when using multiple populations. Across all 11 populations the same small set of haplotypes was found with consistently replicated effects of the haplotypes on androstenone, making the results very robust and useful for breeding programs selecting animals with reduced androstenone level.

### RNA-seq analysis

From the six genes that were differentially expressed in the liver and testis, *SULT2A1* is an obvious candidate gene as it is involved in the metabolism of steroids. This gene is a sulfotransferase enzyme which sulfoconjugates α-androstenone. Increased expression of *SULT2A1* in the testis was found in the pool of animals with high-androstenone haplotype 2 (Table 1).

The higher level of *SULT2A1* in the testis was associated with higher androstenone level in fat tissue. This was unexpected based on the predictions by Sinclair & Squires [5] that animals with low ability to sulfoconjugate 5α-androstenone in the testis would have higher accumulation of this hormone in fat tissue. Nevertheless, three other studies on different breeds (Duroc, Norwegian Landrace, and Yorkshire) [13-15] are in accordance with our results, showing up-regulation of *SULT2A1* in the testis of high-androstenone animals. Androstenone is known to be sulfoconjugated in the testis [5], presumably to facilitate excretion and subsequent transport as androstenonesulfate in the blood. As suggested by Moe et al. [15], high androstenone levels might induce an increase in *SULT2A1* expression in the testis. Recent results suggest, however, that *SULT2A1* might not be involved in the sulfoconjugation of androstenone and that another sulfotransferase is involved in this step, or that it is involved only in combination with enolase [25]. Moe et al. [26] also studied gene expression in the liver and found many genes to be differentially expressed but not *SULT2A1*, similar to our observation for the liver.

Another candidate gene that was differentially expressed in the testis is *FTL*. The *FTL* gene codes for the ferritin light chain, an iron storage protein involved in numerous essential cellular functions. Although the function of *FTL* in the synthesis of androstenone has not been investigated [14], it was suggested by Moe et al. [15] that *FTL* may influence androstenone level by interaction with *CYB5A* that may affect the *CYB5/CYP450* electron transfer. As the role of *FTL* affecting androstenone has not been investigated in more detail and in our study we did not find any variants that could explain a difference in expression, it remains unclear whether it has a direct effect on androstenone level. It was, therefore, not considered to be a strong candidate gene. Our expression data for *FTL* is consistent with the findings of three other studies [13-15], where *FTL* was up-regulated in Duroc, Norwegian Landrace, and Yorkshire boars with high androstenone levels.

### Functional analysis using DNA sequence data

#### Coding regions

The only gene within the 1.94 Mbp region for which a non-synonymous variation was identified between haplotypes 1 and 2 that might have an impact on protein function was *FUT1. FUT1* has been identified as a candidate gene controlling the adhesion of enterotoxigenic *Escherichia coli* (ETEC) F18 to the F18 receptor [27]. However, *FUT1* is not known to have an influence on androstenone level, and based on the functions of the protein encoded by this gene, it is unlikely that it affects androstenone level.

#### Regulatory regions

We studied the regulatory regions of *SULT2A1* and *FTL* because they were the two candidate genes that were differentially expressed according to the RNA-seq analysis. We checked potential TFBSs and CpG islands, and only one variation (C/G, 49,110,873 bp) was found within a CpG island (49,110,687 bp - 49,110,889 bp) predicted for *SULT2A1*.

CpG islands are known to play a role in regulating gene expression where, in general, higher methylation levels are related to repression of gene expression [28]. This one variation found within the CpG island could explain the difference in expression of *SULT2A1* caused by the haplotype, however, this difference in expression between haplotypes identified by RNA-seq could not be validated subsequently, making it very unlikely that this variation plays a role in gene regulation.

### Validation of differential *SULT2A1* expression

Allele-specific expression analysis was a follow-up step to the RNA-seq experiment to test the association of the haplotypes with difference in the ratio of *C*:*T* expression of *SULT2A1* within heterozygous animals. The quantitative difference in the relative expression found for RNA-seq (2.5:1) and allele-specific expression analysis (1.5:1) may simply be due to random error in the estimate from RNA-seq analysis which was based on only two pooled samples. Other reasons include systematic or technical differences that affect the amplification in the RNA-seq assay. There may be other biological mechanisms that trigger a higher expression of *SULT2A1* allele *C* that cannot be captured by allele-specific expression analysis. Unraveling such a mechanism can however not be achieved using our data. Surprisingly, in the allele-specific expression analysis we did not observe differential expression between heterozygous animals (C/T) with either low/low or high/low androstenone diplotypes (Figure 4). We concluded that the difference in *SULT2A1* expression was not regulated by the haplotypes surrounding the *SULT2A1* gene. Instead, an increase in expression of allele *C* over allele *T* in *SULT2A1* was observed, indicating haplotype independent allelic-imbalanced expression between these two alleles. One option for the cause of this allelic-imbalanced expression is a potential regulatory SNP-variant in LD with the *SULT2A1* SNP that affects expression. Other options are transcriptional regulation of the two alleles, like in an enhancer element, that resides outside the investigated region, or differences in RNA decay between the two alleles. It is known that the RNA folding structures play a role in the degree of RNA decay. Prediction of the fold structure indicated a considerable difference in structure around the two alleles (Madsen, O., unpublished observation) making RNA decay a possible participant in the observed allelic-imbalanced expression.

### Origin of the haplotypes

Since the entire region between 48.3 Mbp and 50.2 Mbp on SSC6 has a very low recombination rate [24], the integrity of the haplotypes found in this study has been retained across different populations. Because of this retained integrity, a phylogenetic analysis could be applied to construct a phylogenetic tree of this region from sequencing data from the 55 sequenced animals (Figure 5) [23].

This tree revealed that haplotype 1 of the 1.94 Mbp region, associated with low androstenone level, originated from Asia. It is likely, therefore, that haplotype 1 was introgressed into European breeds during the 18^th^ and 19^th^ centuries, generating hybrid European breeds [29]. Introgression of favorable Asian haplotypes has been observed for other traits as well. A well-known example is an *IGF2* haplotype conferring increased muscle mass and leaner pigs [30]. This haplotype is currently in high frequency in several commercial pig populations, but originated from Asian pigs. There is currently only a handful of gene variants described from European pigs that originate from the late 18^th^- early 19^th^ century introgression of Asian breeding stock (e.g. [31]).

The likely relatively recent (i.e., around 200 years ago or less) introgression of the Asian haplotypes into the European pigs, combined with the very low recombination rate in the genomic region, further explains the paucity of recombinant haplotypes, and difficulty in fine-mapping even across breeds.

Pigs with Asian origin haplotypes were associated with low-androstenone level, whereas European-origin haplotypes were associated with high androstenone level. This is consistent with Lee et al. [32] who found that Large White alleles have an additive effect on androstenone level for a QTL found on SSC6 at 91 cM, between SW782 (49,996,734 bp-49,996,825 bp) and SW1823 (79,653,393 bp-79,653,597 bp), in an F2 Large White x Meishan population.

Taking into account that haplotypes of European breeds originated from Asian breeds and that Asian breeds have high genetic diversity [33], further studies are needed either to identify additional haplotypes that are recombinant between European and Asian animals or to fine-map the region further in Asian pigs since LD will be much lower than in European pigs.

## Conclusions

In summary, the androstenone QTL region previously identified on SSC6 [9] was narrowed down from 3.75 Mbp to 1.94 Mbp. Differential expression was observed for six genes by RNA-seq analysis. No difference in the ratio of *C*:*T* expression of *SULT2A1* for the haplotypes was found by the allele-specific expression analysis, however, a difference in expression between the *C* over *T* allele was found for a variation within *SULT2A1*, showing that the difference in androstenone levels between the haplotypes is not caused by the SNP in exon 2. Nonetheless, a difference in ln-androstenone level across populations in case of fixation of the Asian-origin haplotype 1 would yield a change, on average, of −0.19 ln-androstenone (ranging from −0.57 to +0.08). Use of tag-SNPs from the haplotype-1 group will be valuable in animal breeding programs to select animals with lower androstenone levels.

## Methods

This study was conducted according to regulations of Dutch law on protection of animals.

### Phenotypes, animals, and genotypes

Phenotypes for androstenone level were obtained from 2,750 boars slaughtered at a mean hot carcass weight of 91.33 kg (SD = 9.21 kg). Androstenone level was measured in fat samples; details of measurements and fat extraction are described in earlier studies [9,34]. Androstenone level was log-transformed (ln-androstenone) because it was not normally distributed. Boars belonged to six purebred populations (population 1 to 6, Duroc-based, Yorkshire-based, Dutch Landrace, Pietrain, Finish Landrace, and Large White) and five terminal crosses based on populations 1–6 (population 7 to 11). Number of animals per population ranged from 940 for a Duroc-based population to 69 for one of the crosses (Table 2).

**Table 2:** Number of animals and means (standard deviation) for ln-androstenone and androstenone (μg/g) per population

**Population**	**Number of animals**	**Ln-androstenone (SD)**	**Androstenone μg/g (SD)**
1	940	0.24 (0.89)	1.84 (1.62)
2	295	−0.31 (0.83)	1.02 (0.91)
3	208	−0.04 (0.83)	1.33 (1.21)
4	207	−1.29 (0.99)	0.45 (0.49)
5	169	0.25 (0.94)	1.88 (1.64)
6	107	0.17 (1.15)	2.14 (2.46)
7	325	−0.61 (0.87)	0.82 (0.96)
8	275	−0.12 (0.88)	1.31 (1.29)
9	83	0.03 (0.82)	1.43 (1.25)
10	72	0.34 (0.94)	2.18 (2.37)
11	69	0.19 (0.83)	1.67 (1.45)

Genotyping was performed using the Illumina PorcineSNP60 BeadChip [8]. Quality control involved removing SNPs with low quality score (GenCall score <0.7), and those with a minor allele frequency lower than 0.01 [9]. A total of 3,025 SNPs located on SSC6 remained and 46 were used in the analyses.

### Linkage Disequilibrium (LD) analysis

Significant SNPs (N = 29) previously identified [9] were rearranged according to the Sscrofa10.2 reference genome and LD blocks were defined based on the criteria of Gabriel et al. [35].

### Haplotype diversity

Haplotypes with frequencies greater than 1% across all populations were identified using Haploview 4.2 [36]. A phylogenetic tree for haplotypes based on the similarities among the 46 SNPs from the haplotypes was constructed using the neighbor-joining method as implemented in MEGA 5 [17].

### Association analysis

Phasing and imputation of sporadic missing data were performed using BEAGLE [37].

Linear regression was used to estimate effect of haplotypes for each population using ASReml v3.0 [38]. The following model was used

$$y_{i}=b_{1}\mathrm{haplo}+a_{i}+e_{i}$$

where y_i_ is the ln-androstenone of the i^th^ animal; b_1_ is the regression coefficient on the haplotype; a_i_ is the random additive genetic effect of the i^th^ animal; e_i_ is the random residual effect.

For the posterior analysis using three haplotypes, only populations that had the third haplotype that was being compared to haplotypes 1 and 2 were included in the analysis. In this analysis, the model was corrected for population.

To test whether groups of haplotypes that are on different branches of the phylogenetic tree have a statistically significant different effect on the androstenone level, we used Treescan which “cuts” the haplotype tree at different branch points to identify functional grouping of haplotypes.

### DNA sequence data

The sequencing procedure used for the 55 sequenced individuals in the present study was described in Bosse et al. [23]. Briefly, Illumina-formatted (v. 1.3-1.7) fastq files, with sequence reads of 100 bp (Illumina HiSeq2000), were subject to quality trimming prior to sequence alignment. A minimum average quality score of 13 (i.e., average error probability equal to 0.05) in a 3 bp window was used as cut-off, with 3-prime sequences being discarded if the criterion was not met. Only sequences where both mates were at least 45 bp in length were retained.

Sequences were aligned against the Sscrofa10.2 reference genome using Mosaik align v.1.1.0017 (https://github.com/wanpinglee/MOSAIK). Alignment was performed using a hash-size of 15, with a maximum of 10 matches retained, and 7% maximum mismatch score, for all pig populations and outgroup species. Alignment files were then sorted using the “mosaiksort” function, which entails removing ambiguously mapped reads that are either orphaned or fall outside a computed insert-size distribution.

Variant allele-calling was performed per individual using the “pileup” function in SAMtools v. 1.12a [39], and variations were initially filtered to have minimum quality of 50 for indels, and 20 for SNPs. In addition, all variants showing higher than 3x average read-density, estimated from the number of raw sequence reads, were also discarded to remove false-positive variant-calling originating from off-site mapping as much as possible. This procedure yielded high-quality variants for 55 pigs, wild boars, and outgroup species (European Nucleotide Archive (ENA) under project number ERP001813).

### Phylogenetic analysis

Sequence assemblies for the region on SSC6 between Sscrofa10.2 reference genome base 48,317,509 and 50,259,057 were extracted per individual according to their genomic coordinates from the BAM files generated from individually-sequenced pigs using SAM tools v.1.12a. Phylogenetic analysis was done using RAxML [40], using 10 iterations and implementing a GTR-Г model of sequence evolution, and with an African warthog as outgroup.

### Functional analysis using DNA sequence data

The two haplotypes present across all populations were numbered haplotype 1 and 2 and re-sequencing data were used to identify putative functional differences between them.

To obtain genotype calls for all polymorphic sites identified across the 55 individuals for which whole-genome sequence data were available, every individual was examined for the genotype call for each of the sites found to be polymorphic in the region of interest, including the species-specific differences. Filtering was based on sequence depth (genotype retained if depth ranged between four reads, and twice the average genome-wide depth), where for this procedure the average sequence depth was based directly on the actual sequence depth measured for each individual separately. Further filtering of these sequence-derived genotypes was performed on SNP and consensus quality (for homozygotes, either a SNP or consensus quality > 20 was applied, and for heterozygotes, both SNP and consensus qualities > 20 were applied).

With the SNPs that contributed different alleles to haplotype 1 and 2, we performed an analysis to annotate functional genetic variants detected between the haplotypes using ANNOVAR. For SNPs that resulted in an amino acid substitution we used PolyPhen2 to predict the impact of this substitution on the structure and function of the protein. To identify mutations outside the exonic regions that have the potential to influence androstenone level, we used MULAN [41] to detect potential TFBSs that were conserved across three species (*Sus scrofa*, *Bos taurus*, and *Homo sapiens*). SNPs between haplotypes 1 and 2 were checked for being located within a TFBS. CpG islands were predicted using EMBOSS/CpGPlot with default settings to identify SNPs between haplotypes 1 and 2 that could be located within a CpG island.

### RNA sequencing data and gene expression analysis

Forty-eight animals of the Duroc-based population were slaughtered at a mean age of 173 days, and The liver and testis tissue were collected and stored in RNALater (Qiagen Inc., Valencia, CA, USA). These samples were genotyped for the 29 SNPs that were significant in [9]. The liver and testis were collected for this analysis because androstenone is synthesized in the testis and metabolized in the liver, leading us believe that they are the most interesting tissues to use for the analysis of the effect of gene expression on androstenone levels. Four animals homozygous for the haplotype associated with high androstenone level were selected for RNA isolation in both the liver and testis, as well as four animals homozygous for the haplotype associated with low androstenone level. From these samples, total RNA was extracted with the RNeasy mini kit (Qiagen Inc., Valencia, CA, USA) following manufacturer’s instructions. RNA from low- and high-androstenone haplotypes were pooled, respectively, and stored at -80C until being used. The Illumina mRNA-seq Sample Preparation Kit was used for sample preparation (~5 μg of total RNA) following manufacturer’s instructions and used for 100 bp single-end cDNA sequencing on the Illumina Highseq 2000 platform.

The sequence data obtained from the two RNA pools were clipped to remove adapter sequence and quality trimmed (Phred score > 20) with Trim galore v.0.2.2 (http://www.bioinformatics.babraham.ac.uk/projects/trim_galore). After cleaning the data, between 58 – 62 million sequence reads were available from each pool. To compare gene and transcript expression, we followed the protocol described in Trapnell et al. [42]. Reads were aligned against the Sscrofa10.2 reference genome with TopHat v.1.4.1 [43] using the -T option (all other options were default) in order to align the reads only against an annotated transcriptome. Estimation of differences in expression was done with Cufflinks v.1.3.0 [43] and visualized with the CummeRbund package [44]. Many imperfections exist in the current Sscrofa10.2 reference genome that may affect the details of the gene model, however we do not think that these imperfections compromise our conclusions. The position of *SULT2A1* is not in doubt because the BAC-by-BAC sequencing strategy, based on a rather good physical map, has been shown to result in overall well-assembled genome sequences at a larger scale (e.g. [24]). The most important inconsistencies are within BACs, because BACs were shotgun-sequenced (using classical Sanger sequencing strategies) at low average depth of ~4-6x [16].

Allele-specific expression analysis was performed for the exonic mutation within *SULT2A1* (C/T change within exon 2 at position 166; SSC6: 49,117,861 bp in Sscrofa10.2), on testis tissue from 67 heterozygous animals. We prepared a Taqman PCR Reaction using the assaymix 40× for *SULT2A1* (AHN1RKQ, Applied Biosystems). Taqman PCR was performed on ABI 7500 RT-PCR system. Output values of cycle 40 (exponential phase) were used for both *C* and *T* signals. These values were corrected for background noise by subtracting the value for the respective signal of cycle 1. To certify that the allele-specific expression analysis was accurate, we quantified the two alleles of the SNP in genomic DNA mixes with known ratios: 4:1, 2.33:1, 1.5:1, 1:1, 1:1.5, 1:2.33, 1:4, and in three concentrations of genomic DNA: 2.5 ng, 10 ng, 40 ng. Ratios were a mix of genomic DNA from two homozygous animals for different alleles. For 10 ng and 40 ng dilutions, 13 heterozygous animals were included in the 1:1 class [45].

## Competing interests

The authors declare that they have no competing interests.

## Authors’ contributions

AMH designed the experiment, analyzed the data and drafted the manuscript. JWMB designed the experiment, provided comments and suggestions regarding data analysis, and took part in writing the paper. BH provided comments and suggestions regarding data analysis and the manuscript. HJM analyzed the data and gave valuable input regarding the phylogenetic analysis. OM designed the experiment and gave valuable input regarding RNA-seq and allele-specific expression analysis. RPMAC organized and coordinated lab material and analysis, MAMG provided comments and suggestions regarding the manuscript. All authors read and approved the final manuscript.

## Supplementary Material

Additional file 1 Position of the 46 SNPs in the QTL region on SSC6 of Sscrofa10.2. Click here for file

Additional file 2 Haplotype effects and total number of animals per pig population. Click here for file

Additional file 3 Haplotype effects estimated using only populations that had the third haplotype that was being compared to haplotypes 1 and 2. Click here for file

Additional file 4 **Average heterozygosity for the narrowed region.** Average heterozygosity in 10,000 bp bins along the narrowed region for a heterozygous animal (A) and a homozygous animal (B). Click here for file

Additional file 5 List of genes and the status of the exonic variation. Click here for file

Additional file 6 **The QTL effects for the *C* and *T* allele of the high- and low-androstenone haplotypes.** Click here for file

Additional file 7 **Panels with control allele-specific expression.** Log-transformed (base 2) data relation between observed and expected ratios of mixed genomic DNA from two homozygous animals for different alleles in three concentrations: 2.5 ng, 10 ng, and 40 ng. Click here for file

Additional file 8 Genetic and physical map of the narrowed region (SSC6: 48,317,509 bp – 50,259,057 bp), showing its low-recombining nature. Click here for file
