# Supplementary material for: On the relationship between an Asian haplotype on chromosome 6 that reduces androstenone levels in boars and the differential expression of SULT2A1 in the testis
Source: BMC Genet. 2014 Jan 9;15:4. doi: 10.1186/1471-2156-15-4 (PMC3890517; doi:10.1186/1471-2156-15-4)
Supplement: Additional file 1 — Position of the 46 SNPs in the QTL region on SSC6 of Sscrofa10.2. [file 1471-2156-15-4-S1.doc]

Table S1. Position of the 46 SNPs in the QTL region on SSC6 of Sscrofa10.2.

| SNP name | Position (bp) |
| --- | --- |
| H3GA0053864 | 48585961 |
| ALGA0102689 | 48717238 |
| ASGA0104037 | 48792292 |
| ASGA0089838 | 49146524 |
| ASGA0093393 | 49168322 |
| MARC0019764 | 49351202 |
| MARC0015928 | 49538608 |
| DIAS0000492 | 49802217 |
| MARC0011519 | 49817264 |
| H3GA0056470 | 50006716 |
| DIAS0004447 | 50037571 |
| DIAS0003231 | 50065951 |
| ASGA0084861 | 50079246 |
| MARC0032442 | 50259057 |
| DIAS0000822 | 50264414 |
| H3GA0053555 | 50307537 |
| DIAS0003830 | 50339827 |
| MARC0049189 | 50364492 |
| MARC0044346 | 50478565 |
| M1GA0008536 | 50495796 |
| H3GA0017949 | 50532885 |
| ASGA0028206 | 50556192 |
| M1GA0008527 | 50606084 |
| ASGA0028216 | 50742441 |
| ASGA0028211 | 50803585 |
| M1GA0008539 | 50847065 |
| ASGA0103898 | 50867656 |
| MARC0098482 | 50888554 |
| H3GA0056609 | 50922233 |
| ALGA0122867 | 51104922 |
| ALGA0116613 | 51139647 |
| ASGA0103416 | 51352837 |
| ALGA0035323 | 51611976 |
| ALGA0035324 | 51636474 |
| ALGA0035318 | 51678926 |
| MARC0021351 | 51692785 |
| ASGA0028223 | 51757391 |
| ALGA0035326 | 51775907 |
| ASGA0028228 | 51805308 |
| ALGA0035330 | 51843873 |
| MARC0086794 | 52063034 |
| ALGA0115158 | 52085979 |
| ASGA0097167 | 52127558 |
| ALGA0112704 | 52226606 |
| MARC0005462 | 52262806 |
| MARC0049139 | 52336598 |
