# Supplementary material for: On the relationship between an Asian haplotype on chromosome 6 that reduces androstenone levels in boars and the differential expression of SULT2A1 in the testis
Source: BMC Genet. 2014 Jan 9;15:4. doi: 10.1186/1471-2156-15-4 (PMC3890517; doi:10.1186/1471-2156-15-4)
Supplement: Additional file 2 — Haplotype effects and total number of animals per pig population. [file 1471-2156-15-4-S2.doc]

Table S2. Haplotype effects and total number of animals per pig population

| Haplotype | Pop. 1 | Pop. 2 | Pop. 3 | Pop. 4 | Pop. 5 | Pop. 6 | Pop. 7 | Pop. 8 | Pop. 9 | Pop. 10 | Pop. 11 |
| --- | --- | --- | --- | --- | --- | --- | --- | --- | --- | --- | --- |
| 1 | -0.1873 | -0.0734 | 0.0582 | -0.1530 | -0.0345 | -0.3073 | -0.1512 | -0.0541 | -0.0112 | -0.3086 | -0.1777 |
| 2 | 0.1023 | 0.0422 | 0.1779 | -0.0940 | -0.0628 | 0.1899 | -0.0464 | 0.0057 | 0.0449 | 0.1434 | 0.2384 |
| 3 | -0.0799 | NA | NA | 0.3426 | NA | NA | NA | -0.1811 | NA | -0.2958 | 0.4018 |
| 4 | 0.1025 | NA | NA | NA | 0.0172 | 0.0496 | NA | 0.2317 | -0.0663 | 0.1292 | 0.5532 |
| 5 | NA | 0.2281 | 0.0365 | -0.1396 | 0.1015 | NA | -0.1768 | 0.2509 | -0.2750 | NA | NA |
| 6 | NA | NA | 0.0900 | NA | 0.0734 | NA | 0.0239 | 0.1694 | 0.3014 | 0.6214 | NA |
| 7 | NA | -0.0350 | NA | NA | 0.0492 | 0.0002 | 0.2827 | -0.1139 | NA | NA | -0.3860 |
| 8 | NA | 0.0819 | NA | 0.0495 | NA | NA | NA | NA | -0.0579 | NA | -0.2053 |
| 9 | 0.1542 | NA | NA | NA | NA | 0.3649 | 0.4696 | 0.0815 | -0.0172 | 0.3131 | NA |
| 10 | NA | -0.1542 | -0.0267 | 0.1195 | NA | NA | -0.4075 | -0.3729 | -0.5332 | NA | 0.3084 |
| Total Animals | 940 | 295 | 208 | 207 | 169 | 107 | 325 | 275 | 83 | 72 | 69 |
