# Supplementary material for: On the relationship between an Asian haplotype on chromosome 6 that reduces androstenone levels in boars and the differential expression of SULT2A1 in the testis
Source: BMC Genet. 2014 Jan 9;15:4. doi: 10.1186/1471-2156-15-4 (PMC3890517; doi:10.1186/1471-2156-15-4)
Supplement: Additional file 3 — Haplotype effects estimated using only populations that had the third haplotype that was being compared to haplotypes 1 and 2. [file 1471-2156-15-4-S3.doc]

Table S3. Haplotype effects estimated using only populations that have the third haplotype that is being compared to haplotypes 1 and 2

| Haplotype | Estimate | Std. Error |
| --- | --- | --- |
| Haplotype 3 | | |
| **Haplotype1** | **-0.22** | **0.04** |
| Haplotype2 | -0.01 | 0.04 |
| **Haplotype3** | **-0.15** | **0.05** |
| Haplotype 4 | | |
| Haplotype1 | -0.14 | 0.04 |
| **Haplotype2** | **0.12** | **0.04** |
| **Haplotype4** | **0.16** | **0.05** |
| Haplotype 5 | | |
| Haplotype1 | -0.15 | 0.04 |
| **Haplotype2** | **-0.03** | **0.04** |
| **Haplotype5** | **-0.01** | **0.05** |
| Haplotype 6 | | |
| Haplotype1 | -0.11 | 0.05 |
| **Haplotype2** | **-0.01** | **0.05** |
| **Haplotype6** | **0.10** | **0.06** |
| Haplotype 7 | | |
| Haplotype1 | -0.16 | 0.04 |
| **Haplotype2** | **0.00** | **0.05** |
| **Haplotype7** | **0.05** | **0.07** |
| Haplotype 8 | | |
| Haplotype1 | -0.11 | 0.06 |
| Haplotype2 | 0.03 | 0.07 |
| Haplotype8 | 0.07 | 0.09 |
| Haplotype 9 | | |
| Haplotype1 | -0.15 | 0.04 |
| **Haplotype2** | **0.07** | **0.04** |
| **Haplotype9** | **0.26** | **0.08** |
| Haplotype 10 | | |
| Haplotype1 | -0.15 | 0.04 |
| Haplotype2 | -0.05 | 0.04 |
| Haplotype10 | -0.13 | 0.08 |
