# Supplementary figures and images for: On the relationship between an Asian haplotype on chromosome 6 that reduces androstenone levels in boars and the differential expression of SULT2A1 in the testis
Source: BMC Genet. 2014 Jan 9;15:4. doi: 10.1186/1471-2156-15-4 (PMC3890517; doi:10.1186/1471-2156-15-4)

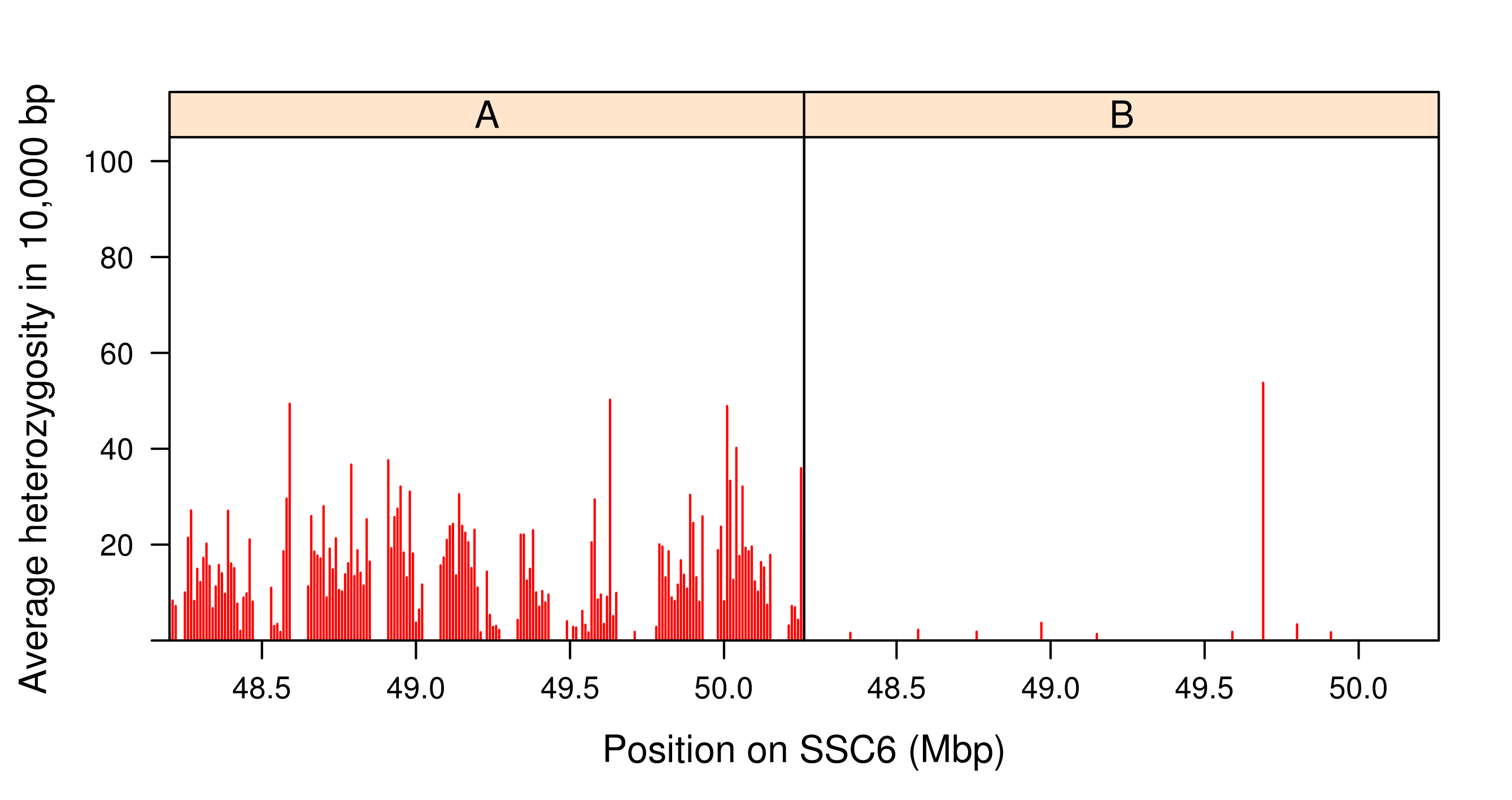

Supplement: Additional file 4 — Average heterozygosity for the narrowed region. Average heterozygosity in 10,000 bp bins along the narrowed region for a heterozygous animal (A) and a homozygous animal (B). [file 1471-2156-15-4-S4.tiff]

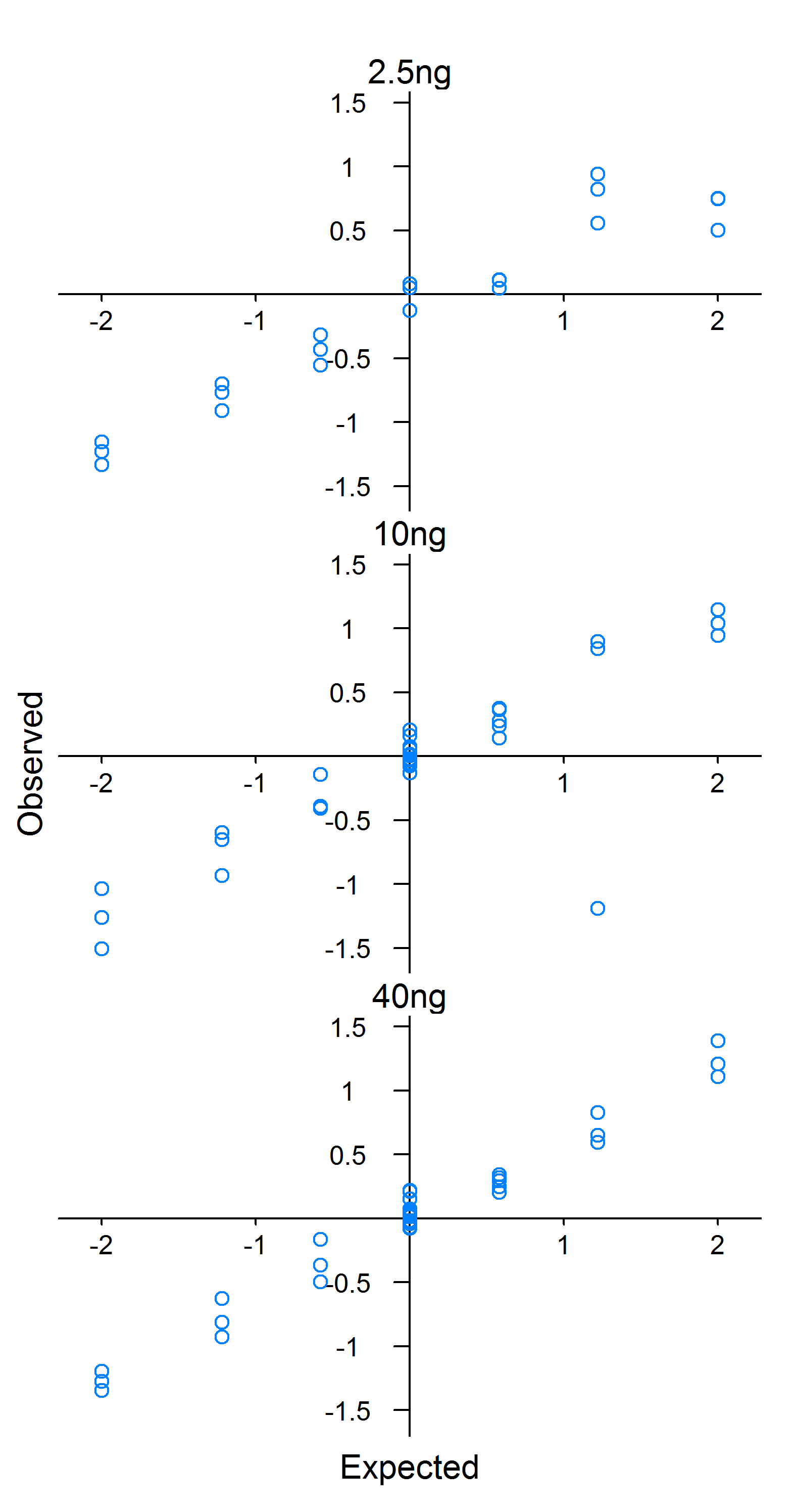

Supplement: Additional file 7 — Panels with control allele-specific expression. Log-transformed (base 2) data relation between observed and expected ratios of mixed genomic DNA from two homozygous animals for different alleles in three concentrations: 2.5 ng, 10 ng, and 40 ng. [file 1471-2156-15-4-S7.tiff]
