# Supplementary material for: On the relationship between an Asian haplotype on chromosome 6 that reduces androstenone levels in boars and the differential expression of SULT2A1 in the testis
Source: BMC Genet. 2014 Jan 9;15:4. doi: 10.1186/1471-2156-15-4 (PMC3890517; doi:10.1186/1471-2156-15-4)
Supplement: Additional file 5 — List of genes and the status of the exonic variation. [file 1471-2156-15-4-S5.doc]

Table S4. List of genes and the status of the exonic variation

| **Gene** | **Exon** | **Status** |  | **Gene** | **Exon** | **Status** |
| --- | --- | --- | --- | --- | --- | --- |
| *GPR77* | Exon1 | Synonymous |  | *MAMSTR* | Exon2 | Synonymous |
| *GPR77* | Exon1 | Synonymous |  | *IZUMO1* | Exon1 | Non-synonymous |
| *GPR77* | Exon1 | Synonymous |  | *FUT1* | Exon1 | Non-synonymous |
| *DHX34* | Exon2 | Synonymous |  | *HSD17B14* | Exon9 | Synonymous |
| *DHX34* | Exon9 | Synonymous |  | *HSD17B14* | Exon4 | Non-synonymous |
| *DHX34* | Exon15 | Synonymous |  | *PLEKHA4* | Exon17 | Synonymous |
| *DHX34* | Exon16 | Synonymous |  | *PLEKHA4* | Exon6 | Synonymous |
| *ZNF541* | Exon1 | Synonymous |  | *PPP1R15A* | Exon1 | Synonymous |
| *ZNF541* | Exon3 | Synonymous |  | *PPP1R15A* | Exon2 | Non-synonymous |
| *ZNF541* | Exon3 | Synonymous |  | *PPP1R15A* | Exon2 | Synonymous |
| *ZNF541* | Exon5 | Synonymous |  | *HRC* | Exon1 | Non-synonymous |
| *ZNF541* | Exon15 | Non-synonymous |  | *HRC* | Exon1 | Non-synonymous |
| *KPTN* | Exon10 | Synonymous |  | *PPFIA3* | Exon26 | Synonymous |
| *SLC8A2* | Exon1 | Synonymous |  | *PPFIA3* | Exon25 | Non-synonymous |
| *SLC8A2* | Exon1 | Synonymous |  | *PPFIA3* | Exon21 | Synonymous |
| *MEIS3* | Exon1 | Synonymous |  | *PPFIA3* | Exon15 | Non-synonymous |
| *MEIS3* | Exon10 | Non-synonymous |  | *PPFIA3* | Exon14 | Non-synonymous |
| *GLTSCR1* | Exon9 | Synonymous |  | *PPFIA3* | Exon13 | Non-synonymous |
| *GLTSCR1* | Exon12 | Synonymous |  | *PPFIA3* | Exon13 | Non-synonymous |
| *GLTSCR1* | Exon13 | Synonymous |  | *PPFIA3* | Exon3 | Synonymous |
| *EHD2* | Exon2 | Synonymous |  | *LIN7B* | Exon3 | Synonymous |
| *GLTSCR2* | Exon1 | Synonymous |  | *SNRNP70* | Exon5 | Synonymous |
| *GLTSCR2* | Exon5 | Synonymous |  | *SNRNP70* | Exon2 | Synonymous |
| *GLTSCR2* | Exon8 | Non-synonymous |  | *KCNA7* | Exon1 | Synonymous |
| *GLTSCR2* | Exon10 | Synonymous |  | *KCNA7* | Exon2 | Synonymous |
| *CRX* | Exon3 | Synonymous |  | *KCNA7* | Exon1 | Synonymous |
| *CRX* | Exon3 | Synonymous |  | *NTF4* | Exon1 | Synonymous |
| *SULT2A1* | Exon2 | Synonymous |  | *LHB* | Exon1 | Synonymous |
| *LIG1* | Exon14 | Synonymous |  | *RUVBL2* | Exon13 | Synonymous |
| *TMEM143* | Exon5 | Synonymous |  | *GYS1* | Exon5 | Synonymous |
| *LMTK3* | Exon5 | Synonymous |  | *FTL* | Exon4 | Synonymous |
| *LMTK3* | Exon11 | Synonymous |  | *NUCB1* | Exon5 | Synonymous |
| *CYTH2* | Exon4 | Synonymous |  | *TULP2* | Exon6 | Non-synonymous |
| *GRWD1* | Exon6 | Synonymous |  | *TULP2* | Exon8 | Non-synonymous |
| *GRIN2D* | Exon11 | Synonymous |  | *TULP2* | Exon10 | Non-synonymous |
| *GRIN2D* | Exon11 | Synonymous |  | *SLC17A7* | Exon10 | Synonymous |
| *GRIN2D* | Exon4 | Synonymous |  | *SLC17A7* | Exon7 | Synonymous |
| *KDELR1* | Exon4 | Synonymous |  |  |  |  |
