# Supplementary material for: On the relationship between an Asian haplotype on chromosome 6 that reduces androstenone levels in boars and the differential expression of SULT2A1 in the testis
Source: BMC Genet. 2014 Jan 9;15:4. doi: 10.1186/1471-2156-15-4 (PMC3890517; doi:10.1186/1471-2156-15-4)
Supplement: Additional file 6 — The QTL effects for the C and T allele of the high- and low-androstenone haplotypes. [file 1471-2156-15-4-S6.doc]

Table S5. The QTL effects for the C and T allele of the high- and low-androstenone haplotypes

| Allele (haplotype) | Estimate | Std. Error |
| --- | --- | --- |
| *C* allele (high-androstenone haplotype) | 0.00 | 0.00 |
| ***T* allele (low-androstenone haplotype)** | **-0.44** | **0.11** |
| ***C* allele (low-androstenone haplotype)** | **-0.29** | **0.11** |
