# Supplementary material for: On the relationship between an Asian haplotype on chromosome 6 that reduces androstenone levels in boars and the differential expression of SULT2A1 in the testis
Source: BMC Genet. 2014 Jan 9;15:4. doi: 10.1186/1471-2156-15-4 (PMC3890517; doi:10.1186/1471-2156-15-4)
Supplement: Additional file 8 — Genetic and physical map of the narrowed region (SSC6: 48,317,509 bp – 50,259,057 bp), showing its low-recombining nature. [file 1471-2156-15-4-S8.doc]

Table S6. Genetic and physical map of the narrowed region (SSC6: 48,317,509 bp – 50,259,057 bp), showing its low-recombining nature

| SNP | SSC | Marker Order | cM | Position |
| --- | --- | --- | --- | --- |
| H3GA0053864 | 6 | 623 | 62.263 | 48585961 |
| ASGA0104037 | 6 | 624 | 62.276 | 48792292 |
| ALGA0102689 | 6 | 625 | 62.290 | 48717238 |
| ASGA0089838 | 6 | 626 | 62.303 | 49146524 |
| MARC0015928 | 6 | 627 | 62.317 | 49538608 |
| MARC0011519 | 6 | 628 | 62.330 | 49817264 |
| DIAS0000492 | 6 | 629 | 62.344 | 49802217 |
| DIAS0004447 | 6 | 630 | 62.357 | 50037571 |
| ASGA0084861 | 6 | 631 | 62.371 | 50079246 |
| DIAS0000822 | 6 | 632 | 62.378 | 50264414 |
| MARC0032442 | 6 | 633 | 62.386 | 50259057 |
| MARC0049189 | 6 | 634 | 62.393 | 50364492 |
| H3GA0053555 | 6 | 635 | 62.401 | 50307537 |
| MARC0044346 | 6 | 636 | 62.408 | 50478565 |
| M1GA0008536 | 6 | 637 | 62.415 | 50495796 |
| H3GA0017949 | 6 | 638 | 62.421 | 50532885 |
| ASGA0028206 | 6 | 639 | 62.428 | 50556192 |
| M1GA0008527 | 6 | 640 | 62.434 | 50606084 |
| ASGA0028211 | 6 | 641 | 62.441 | 50803585 |
| M1GA0008539 | 6 | 642 | 62.447 | 50847065 |
| ASGA0103898 | 6 | 643 | 62.453 | 50867656 |
| H3GA0056609 | 6 | 644 | 62.460 | 50922233 |
| ALGA0122867 | 6 | 645 | 62.466 | 51104922 |
| ALGA0116613 | 6 | 646 | 62.476 | 51139647 |
| ASGA0103416 | 6 | 647 | 62.485 | 51352837 |
| ALGA0035318 | 6 | 648 | 62.495 | 51678926 |
| MARC0021351 | 6 | 649 | 62.504 | 51692785 |
| ASGA0028223 | 6 | 650 | 62.511 | 51757391 |
| ASGA0028228 | 6 | 651 | 62.517 | 51805308 |
| ALGA0035330 | 6 | 652 | 62.524 | 51843873 |
| MARC0086794 | 6 | 653 | 62.530 | 52063034 |
| ALGA0115158 | 6 | 654 | 62.537 | 52085979 |
| ASGA0097167 | 6 | 655 | 62.543 | 52127558 |
| MARC0049139 | 6 | 656 | 62.549 | 52336598 |
| MARC0005462 | 6 | 657 | 62.554 | 52262806 |
| ALGA0112704 | 6 | 658 | 62.559 | 52226606 |
